# JAK2 Inhibition Impairs Proliferation and Sensitises Cervical Cancer Cells to Cisplatin-Induced Cell Death

**DOI:** 10.3390/cancers11121934

**Published:** 2019-12-04

**Authors:** Ethan L. Morgan, Andrew Macdonald

**Affiliations:** School of Molecular and Cellular Biology, Faculty of Biological Sciences and Astbury Centre for Structural Molecular Biology, University of Leeds, Leeds LS2 9JT, UK; e.l.morgan@leeds.ac.uk

**Keywords:** JAK2, STAT3, STAT5B, HPV, cervical cancer

## Abstract

Persistent infection with high-risk human papillomavirus (HPV) is the underlying cause of ~5% of all human cancers, including the majority of cervical carcinomas and many other ano-genital and oral cancers. A major challenge remains to identify key host targets of HPV and to reveal how they contribute to virus-mediated malignancy. The HPV E6 oncoprotein aberrantly activates the signal transducer and activator of transcription 3 (STAT3) transcription factor and this is achieved by a virus-driven increase in the levels of the pro-inflammatory cytokine interleukin-6 (IL-6) in HPV positive cervical cancers cells. Crucially, STAT3 activity is essential for the proliferation and survival of cervical cancer cells, suggesting that targeting STAT3 may have therapeutic potential. Unfortunately, the development of direct STAT3 inhibitors has been problematic in the clinic due to toxicity issues identified in early stage trials. To overcome this issue, we focused on the protein Janus kinase 2 (JAK2), which phosphorylates STAT3 and is essential for STAT3 activation. Here, we demonstrate that inhibiting JAK2 reduces cell proliferation and induces apoptosis in HPV transformed cervical cancer cells. We further establish that this is due to inhibition of phosphorylation of the JAK2 substrates STAT3 and STAT5. Finally, we demonstrate that the clinically available JAK2 inhibitor Ruxolitinib synergises with cisplatin in inducing apoptosis, highlighting JAK2 as a promising therapeutic target in HPV-driven cancers.

## 1. Introduction

High-risk human papillomavirus (HR-HPV) infection accounts for ~5% of human cancer cases worldwide [1,2], causing the majority of cervical cancers (>99%) and between 30–70% of oropharyngeal cancers [3]. HR-HPVs encode the E6 and E7 oncoproteins, which promote cell cycle progression [4], impair host apoptosis and immune activation pathways and enable an accumulation of genomic instability through activation of the DNA damage response [5,6].

Despite the availability of vaccines against HPV infection, these are preventative and there are currently no specific therapeutics for HPV-associated cancers [7]. Thus, the most common treatment for cervical cancer remains generic platinum-based chemotherapeutics such as cisplatin, either alone or combined with radiotherapy [8]. Unfortunately, many cancers develop resistance to these drugs [9] and, as such, there exists a need to identify novel targets for the treatment of HPV-mediated malignancies.

The transcription factor STAT3 is aberrantly activated in many types of cancer [10,11,12,13], and this hyperactivation is usually associated with a poor clinical prognosis. STAT3 is activated by a range of stimuli, including growth factors and cytokines [14], through receptor tyrosine kinases (RTKs) such as the epidermal growth factor receptor (EGFR) and cytokine receptors such as the Interleukin-6 receptor (IL-6R) [11,15,16]. STAT3 can then be activated through phosphorylation at tyrosine 705 (Y705) either directly via receptors such as EGFR or by non-receptor tyrosine kinases such as Src or Janus Kinase 2 (JAK2) [14,17,18]. Phosphorylation of STAT3 induces its dimerisation and nuclear translocation, leading to DNA binding and the induction of STAT3-dependent gene transcription [10,17].

Despite the importance of STAT3 as an oncogenic driver in many cancers, targeting STAT3 therapeutically has been challenging [19]. Therefore, targeting upstream activators of STAT3 may have therapeutic potential.

We have previously demonstrated that inhibition of STAT3 results in the inhibition of proliferation and the induction of apoptosis in HPV positive (HPV+) cervical cancer cells [20]. Here, we demonstrate that JAK2 is aberrantly phosphorylated in cervical cancer cells and that inhibition of JAK2 results in the loss of STAT3 phosphorylation. Furthermore, we show that JAK2 inhibition also reduces the phosphorylation of the related transcription factor STAT5. Inhibition of either JAK2 or STAT5 results in impaired proliferation in HPV+ cervical cancer cells and the induction of apoptosis. Finally, we demonstrate that the clinically available inhibitor Ruxolitinib synergises with cisplatin in the induction of apoptosis in cervical cancer cells, suggesting that pharmalogical targeting of JAK2 may have therapeutic benefit in HPV-driven malignancies.

## 2. Results

### 2.1. JAK2 Is Aberrantly Phosphorylated in Cervical Disease and HPV+ Cervical Cancer Cells

**Figure 1:**
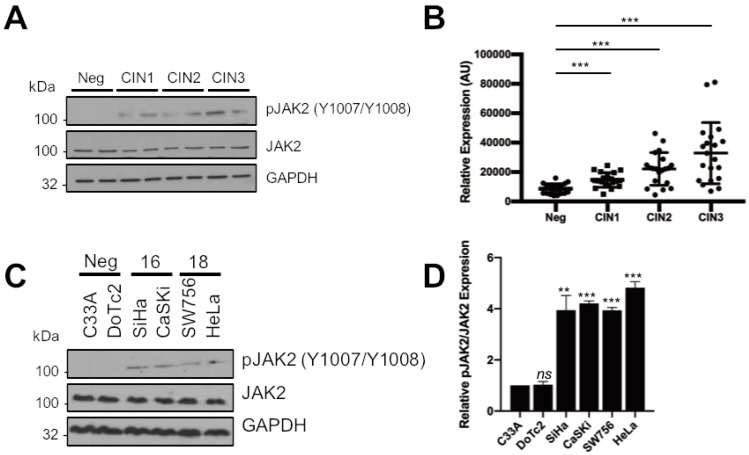
JAK2 is aberrantly phosphorylated in cervical disease and HPV+ cervical cancer cells. (**A**) Representative western blots from cytology samples of CIN lesions of increasing grade analysed for phosphorylated JAK2 and total JAK2 expression. GAPDH served as a loading control. (**B**) Scatter dot plot of densitometry analysis of a panel of cytology samples. Twenty samples from each clinical grade (neg, CIN I–III) were analysed by western blot and densitometry analysis was performed using ImageJ. (**C**) Representative western blot of from six cervical cancer cell lines—two HPV- (C33A and Dotc2 4510), two HPV16+ (SiHa and CaSKi) and HPV18+ (SW756 and HeLa)—for the expression of phosphorylated and total JAK2. GAPDH served as a loading control. Data are representative of at least three biological independent repeats. (**D**) Densitometry analysis from C. Error bars represent the mean ± standard deviation of a minimum of three biological repeats. ns- not significant, ** *p* < 0.01, *** *p* < 0.001 (Student’s *t*-test).

Targeting STAT3 pharmologically has proven difficult in the clinic thus far [19]. Previously, we demonstrated that HPV activates STAT3 via the tyrosine kinase JAK2 in primary human keratinocytes [21]. To determine whether an analogous signalling pathway functions in HPV-driven cervical disease, we analysed a series of cervical liquid-based cytology samples from a cohort of HPV16 positive patients representing the progression of disease development (CIN1-CIN3) and HPV negative normal cervical tissue controls for levels of phosphorylated and total JAK2 protein. Western blot showed that in normal cervical tissue, the level of JAK2 tyrosine phosphorylation was undetectable (Figure 1A). However, JAK2 phosphorylation, but not total levels of the protein, increased in the disease samples and this significantly correlated with the degree of disease severity (Figure 1B; CIN1, *p* = 6.5 × 10^−5^; CIN2, *p* = 6.6 × 10^−6^; CIN3, *p* = 8.1 × 10^−6^).

To confirm if JAK2 phosphorylation remained elevated in cervical cancer, we performed western blot on lysates from a panel of six cervical cancer cell lines. We observed an increase in JAK2 phosphorylation in lysates from both HPV16 positive (HPV16+; SiHa and CaSKi) and HPV18 positive (HPV18+; SW756 and HeLa) cervical cancer cell lines when compared to HPV negative (HPV−; C33A and DoTc2) cervical cancer cells. Again, the levels of total JAK2 protein remained unchanged (Figure 1C). These data suggest that an increase in JAK2 phosphorylation positively correlates with cervical disease severity and that JAK2 is also aberrantly phosphorylated in HPV+ cervical cancer cells.

### 2.2. JAK2 Is Required for STAT3 Phosphorylation and Proliferation in HPV+ Cervical Cancer Cells

**Figure 2:**
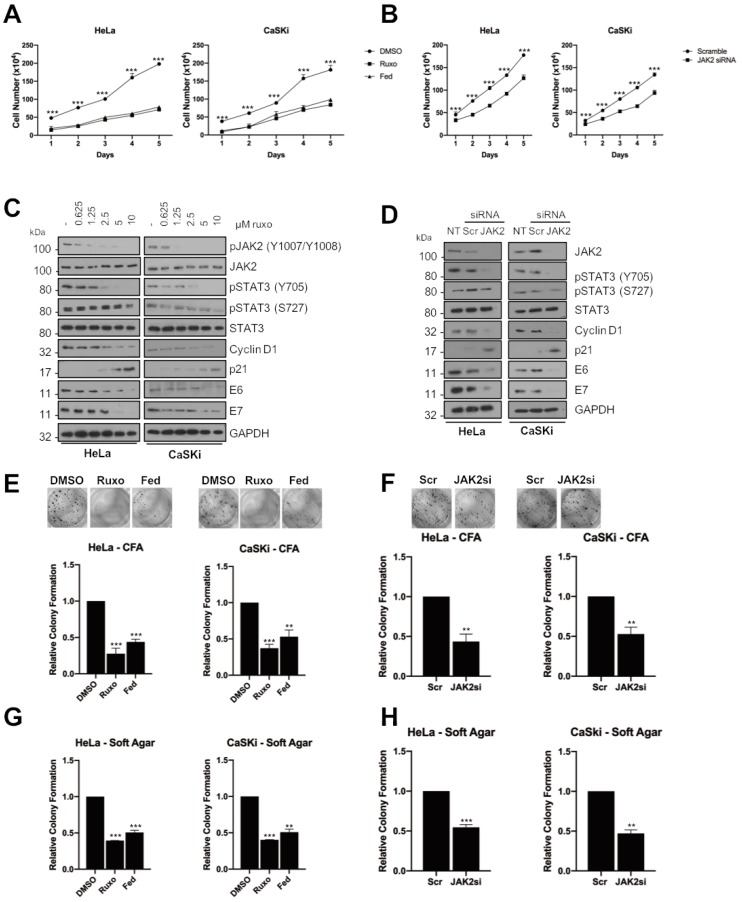
JAK2 is required for STAT3 phosphorylation and proliferation in HPV+ cervical cancer cells. (**A**) Growth curve analysis of HeLa (left) and CaSKi (right) cells after addition of inhibitors for 48 h. (**B**) Growth curve analysis of HeLa (left) and CaSKi (right) after transfection of a pool of four specific JAK2 siRNA for 72 h. (**C**) Representative western blot of ruxolitinib dose response in HeLa and CaSKi cells after 48 h. Densitometry analysis is in Supplementary Figure S3A. (**D**) Representative western blot of HeLa and CaSKi cells after transfection of a pool of four specific JAK2 siRNA for 72 h. Densitometry analysis is in Supplementary Figure S3B. (**E**) Colony formation assay (anchorage dependent growth) of HeLa and CaSKi cells after addition of inhibitors for 48 h. (**F**) Colony formation assay (anchorage dependent growth) of HeLa and CaSKi cells after transfection of a pool of four specific JAK2 siRNA for 72 h. (**G**) Soft agar assay (anchorage independent growth) of HeLa and CaSKi cells after addition of inhibitors for 48 h. (**H**) Soft agar assay (anchorage independent growth) of HeLa and CaSKi cells after transfection of a pool of four specific JAK2 siRNA for 72 h. Error bars represent the mean ± standard deviation of a minimum of three biological repeats. ** *p* < 0.01, *** *p* < 0.001 (Student’s *t*-test).

Our previous work demonstrated that the blockade of STAT3 activity with small molecule inhibitors or siRNA depletion of STAT3 protein adversely affected HPV+ cervical cancer cell proliferation and resulted in an increase in apoptosis [20]. To assess if JAK2 activity was required for this proliferation, we investigated the effect of JAK2 inhibition as an indirect mechanism to impair STAT3 activity. Ruxolitinib (ruxo) is a potent JAK1/2 inhibitor used for the treatment of high-risk myelofibrosis and Fedratinib (fed) is a JAK2 specific inhibitor currently in Phase III trials for high-risk myelofibrosis [22]. Both inhibitors cause a loss of JAK2 activation, as judged by a reduction in JAK2 autophosphorylation at Y1007/Y1008 at the dose used (Supplementary Figure S1A). In both HPV18+ Hela and HPV16+ CaSKi cells, inhibition of JAK2 activation resulted in a significant decrease in cell proliferation (Figure 2A; HeLa, *p* = 0.0007 for ruxolitinib, *p* = 0.001 for fed at day 5; CaSKi, *p* = 0.001 for ruxolitinib, *p* = 0.005 for fedratinib at day 5). To confirm that the pharmacological inhibition of JAK2 resulted in a decrease in STAT3 phosphorylation, cells were treated with increasing concentrations of ruxolitinib and fedratinib. Both inhibitors lead to a dose-dependent reduction of JAK2 phosphorylation (Figure 2C and Supplementary Figure S1B). Importantly, inhibition of JAK2 also led to a dose-dependent reduction in STAT3 tyrosine phosphorylation, whilst having only a minimal effect on STAT3 serine phosphorylation, which is independent of JAK, at the higher doses. JAK2 inhibition caused a reduction in expression of cyclin D1 corresponding with an increase in expression of the cell cycle checkpoint protein p21, consistent with our previous results showing that the expression of these gene products is dependent on STAT3 in HPV+ cells [20,21]. As for our previous studies with STAT3 inhibition, JAK2 inhibition also resulted in a reduction in HPV E6 and E7 expression [20]. Phenotypically, inhibition of JAK2 resulted in a significant decrease in the ability of HPV+ cells to form anchorage-dependent (Figure 2E; HeLa, *p* = 0.0002 for ruxolitinib, *p* = 2 × 10^−5^ for fed; CaSKi, *p* = 0.003 for ruxolitinib, *p* = 0.01 for fedratinib) or anchorage-independent colonies (Figure 2G; HeLa, *p* = 6 × 10^−6^ for ruxolitinib, *p* = 0.03 for fedratinib; CaSKi, *p* = 2 × 10^−5^ for ruxolitinib, *p* = 0.07 for fedratinib).

In line with our data showing that JAK2 phosphorylation was lower in HPV− cervical cancer cells, inhibition of JAK2 in HPV− C33A cells resulted in a dose-dependent reduction in JAK2 and STAT3 phosphorylation but had minimal effect on cell growth (Supplementary Figure S2A), anchorage-dependent (Supplementary Figure S2B) and anchorage-independent colony formation (Supplementary Figure S2C). This suggests that the effect of JAK2 inhibition on cell proliferation is specific to HPV+ cervical cancer cells, corresponding with the higher levels of JAK2 phosphorylation in these cells.

Whilst both the inhibitor compounds used target JAK2, small molecule inhibitors often have off target effects. Therefore, we used a validated pool of JAK2-specific siRNAs to confirm the effects observed were through the specific targeting of JAK2. Treatment with siRNA led to ~80% reduction in JAK2 protein expression (Figure 2D) and resulted in a significant reduction in cell proliferation (Figure 2B; HeLa, *p* = 0.0004 at day 5; CaSKi, *p* = 0.0015 at day 5), anchorage-dependent (Figure 2F; HeLa, *p* = 0.0002; CaSKi, *p* = 0.003) and anchorage independent colony formation (Figure 2H; HeLa, *p* = 0.001; CaSKi, *p* = 0.003). Similar to our observations with the JAK2 inhibitor, siRNA depletion of JAK2 also resulted in a reduction in cyclin D1 expression and an increase in p21 protein levels (Figure 2D). These data confirm that JAK2 is required for the proliferation of HPV+ cervical cancer cells and that loss of JAK2 kinase activity or protein generates a phenotype similar to that observed when STAT3 is inhibited in HPV+ cervical cancer cells.

### 2.3. JAK2 Is Required for HPV+ Cervical Cancer Cell Survival

**Figure 3:**
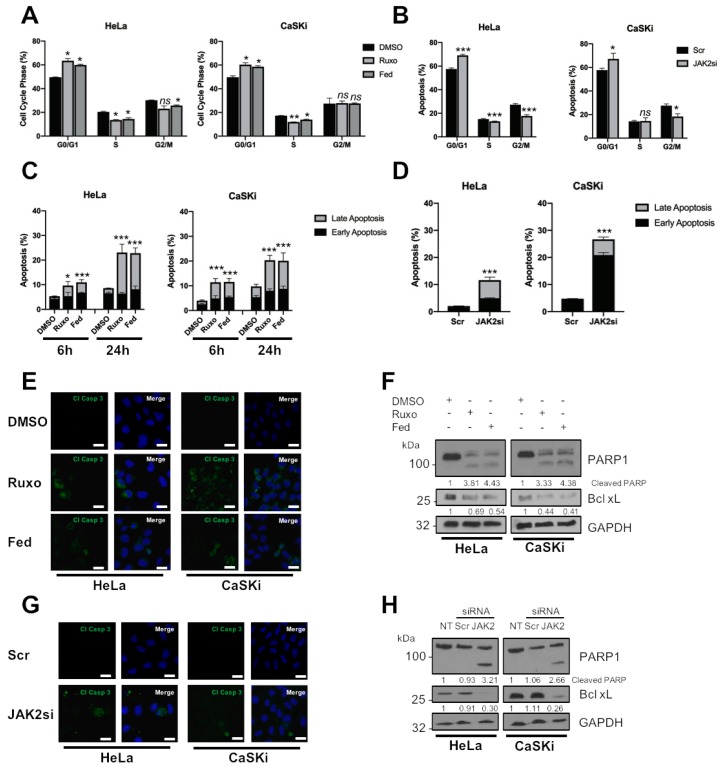
JAK2 is required for HPV+ cervical cancer cell survival. (**A**) Flow cytometric analysis of cell cycle profile of HeLa and CaSKi cells after addition of inhibitors for 24 h. (**B**) Flow cytometric analysis of cell cycle profile of HeLa and CaSKi cells after transfection of a pool of four specific JAK2 siRNA for 72 h. (**C**) Flow cytometric analysis of Annexin V assay in HeLa and CaSKi cells after addition of inhibitors for 6 and 24 h. (**D**) Flow cytometric analysis of Annexin V assay in HeLa and CaSKi cells after transfection of a pool of four specific JAK2 siRNA for 72 h. (**E**) Immunofluorescence analysis of HeLa and CaSKi cells after addition of inhibitors for 6 h. Cover slips were stained for Cleaved caspase 3 (green). Nuclei were visualised using DAPI (blue). Scale bar 20 μM. (**F**) Representative western blot of HeLa and CaSKi cells after addition of inhibitors for 24 h and analysed for PARP cleavage and Bcl xL expression. GAPDH served as the loading control. Densitometry analysis is provided below each blot. (**G**) Immunofluorescence analysis of HeLa and CaSKi cells after transfection of a pool of four specific JAK2 siRNA for 72 h. Cover slips were stained for Cleaved caspase 3 (green). Nuclei were visualised using DAPI (blue). Scale bar 20 μM. (**H**) Representative western blot of HeLa and CaSKi cells after transfection of a pool of four specific JAK2 siRNA for 72 h and analysed for PARP cleavage and Bcl xL expression. GAPDH served as the loading control. Densitometry analysis is provided below each blot. Error bars represent the mean ± standard deviation of a minimum of three biological repeats. ns- not significant, * *p* < 0.05, ** *p* < 0.01, *** *p* < 0.001 (Student’s *t*-test).

JAK2 is able to modulate cell proliferation by controlling the cell cycle in cancer cells through several diverse mechanisms [23,24,25]. We therefore investigated the impact of JAK2 inhibition on the cell cycle in HPV+ cervical cancer cells. We previously demonstrated that STAT3 inhibition resulted in an S-phase block in the cell cycle; in contrast, JAK2 inhibition resulted in a decrease in cells in the S and G2/M phase of the cell cycle and a significant increase of cells in G0/G1 (Figure 3A; HeLa G0/G1, *p* = 0.017 for ruxolitinib, *p* = 0.03 for fedratinib; CaSKi G0/G1, *p* = 0.035 for ruxolitinib, *p* = 0.027 for fedratinib). Similar results were obtained when JAK2 was depleted using JAK2 siRNA (Figure 3B; HeLa G0/G1, *p* = 0.0002; CaSKi G0/G1, *p* = 0.05).

To identify if the block in cell cycle progression and proliferation induced by JAK2 inhibition or depletion resulted in apoptosis, we performed an Annexin V assay. In both HeLa and CaSKi cells, the percentage of early apoptotic cells significantly increased after JAK2 inhibition for 6 h (Figure 3C; HeLa, *p* = 0.09 for ruxolitinib, *p* = 0.001 for fedratinib; CaSKi, *p* = 8 × 10^−5^ for ruxolitinib, *p* =0.003 for fedratinib). By 24 h, the percentage of cells in late apoptosis increased significantly (HeLa, *p* = 0.05 for ruxolitinib, *p* = 0.03 for fedratinib; CaSKi, *p* = 0.03 for ruxolitinib, *p* = 0.05 for fedratinib). Depletion of JAK2 also resulted in a significant increase in apoptosis at 24 h (Figure 3D; total apoptosis, HeLa, *p* = 0.0004; CaSKi, *p* = 1.73 × 10^−5^).

Next, we assessed caspase activation by immunofluorescence analysis and the presence of cleaved poly(ADP) ribose polymerase (PARP), a caspase substrate. Both JAK2 inhibition or depletion resulted in the activation of caspase 3, as determined by the appearance of its cleaved form (Figure 3E,G) and PARP cleavage (Figure 3F,H). The expression of the pro-survival protein Bcl-_XL_, whose expression is dependent on STAT3 in HPV+ cervical cancer cells [20], was also reduced (Figure 3F,H). Together, these data demonstrate that targeting JAK2 using small molecule inhibitors or siRNA results in a G1-arrest of the cell cycle and the induction of apoptosis.

### 2.4. STAT5 Is a Downstream Mediator of JAK2 in HPV+ Cervical Cancer Cells

The above results demonstrate that JAK2 blockade produces a distinct cell cycle phenotype that differs to that observed when STAT3 is inhibited (Figure 3A,B, [20]). Such differences insinuate that loss of an alternative JAK2 substrate may be responsible for the differences in phenotype observed. Of the alternative JAK2 targets studied, the STAT5 transcription factor drew our attention. Firstly, STAT5 is aberrantly activated in several cancers [26,27,28,29], and this is often mediated by enhanced JAK2 activation. Secondly, in addition to STAT3, STAT5 has previously been shown to play an essential role during the productive HPV lifecycle [30,31]. In cervical cancer, STAT5 has been demonstrated to be over-expressed and to correlate with HPV infection [32,33]; however, if STAT5 plays a functional role in cervical cancer pathogenesis is unclear.

**Figure 4:**
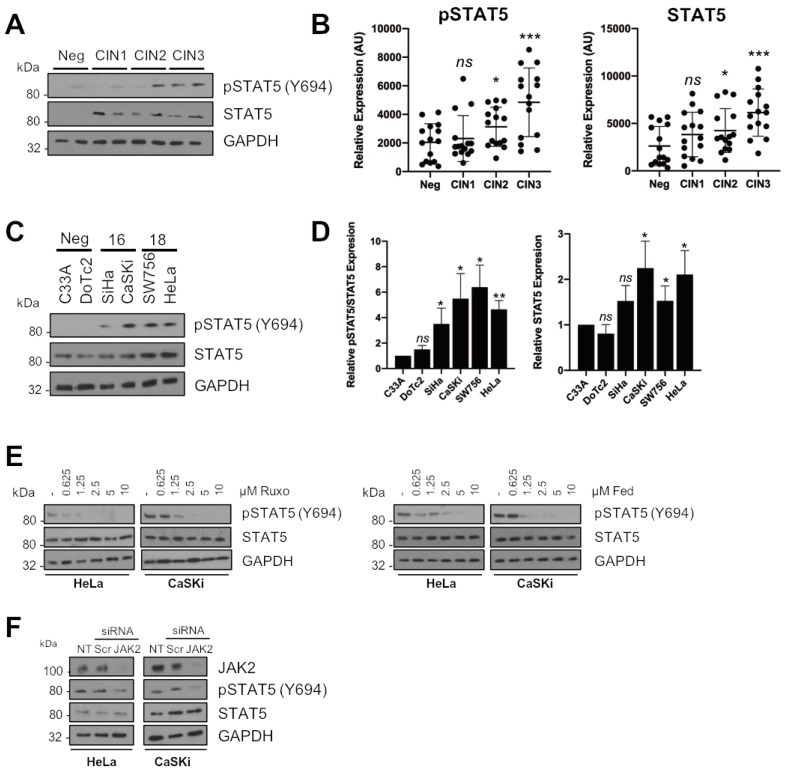
STAT5 is a downstream mediator of JAK2 in HPV+ cervical cancer cells. (**A**) Representative western blots from cytology samples of CIN lesions of increasing grade analysed for phosphorylated STAT5 and total STAT5 expression. GAPDH served as a loading control. (**B**) Scatter dot plot of densitometry analysis of a panel of cytology samples. Twenty samples from each clinical grade (neg, CIN I-III) were analysed by western blot and densitometry analysis was performed using ImageJ. (**C**) Representative western blot of from six cervical cancer cell lines—two HPV- (C33A and Dotc2 4510), two HPV16+ (SiHa and CaSKi) and HPV18+ (SW756 and HeLa)—for the expression of phosphorylated and total STAT5. GAPDH served as a loading control. (**D**) Densitometry analysis from C. (**E**) Representative western blot of ruxolitinib dose response in HeLa and CaSKi cells after 48 h. GAPDH served as a loading control. Densitometry analysis is in Supplementary Figure S4A. (**F**) Representative western blot of HeLa and CaSKi cells after transfection of a pool of four specific JAK2 siRNA for 72 h. GAPDH served as a loading control. Data are representative of at least three biological independent repeats. Densitometry analysis is in Supplementary Figure S4B. Error bars represent the mean ± standard deviation of a minimum of three biological repeats. ns- not significant, * *p* < 0.05, ** *p* < 0.01, *** *p* < 0.001 (Student’s *t*-test).

Therefore, we assessed if STAT5 phosphorylation and/or expression was modulated in HPV+ cervical cancer cells. First, we utilised the cervical liquid-based cytology samples representing pre-cancerous cervical disease progression (CIN1-3) and normal cervical tissue as shown for JAK2. STAT5 phosphorylation increased in line with cervical disease grade from CIN1 to CIN3 (Figure 4A; larger cohort quantified in Figure 4B; CIN1, *p* = 0.62; CIN2, *p* = 0.034; CIN3, *p* = 0.0005). In contrast to JAK2, total STAT5 expression also increases with cervical disease progression (Figure 4A; larger cohort quantified in Figure 4B; CIN1, *p* = 0.14; CIN2, *p* = 0.05; CIN3, *p* = 0.0002).

Additionally, increased STAT5 phosphorylation was observed in both HPV16+ and HPV18+ cervical cancer cell lines when compared to HPV- cervical cancer cells (Figure 4C; quantified in Figure 4D). As with the clinical samples, STAT5 expression was also increased in the HPV+ cell lines (Figure 4C; quantified in Figure 4D). We then assessed if JAK2 was required for the phosphorylation of STAT5 in cervical cancer cells. Firstly, we demonstrated that ruxolitinib or fedratinib treatment reduced STAT5 tyrosine phosphorylation in a dose-dependent manner in HPV+ cervical cancer cells (Figure 4E). Importantly, siRNA depletion of JAK2 similarly reduced STAT5 phosphorylation without effecting total STAT5 expression (Figure 4F). These data suggest that STAT5 is also activated in HPV+ cervical cancer cell lines, and thus, may also contribute to the oncogenic effects of JAK2 in addition to STAT3.

### 2.5. STAT5 Is Required for Proliferation in HPV+ Cervical Cancer Cell Proliferation

**Figure 5:**
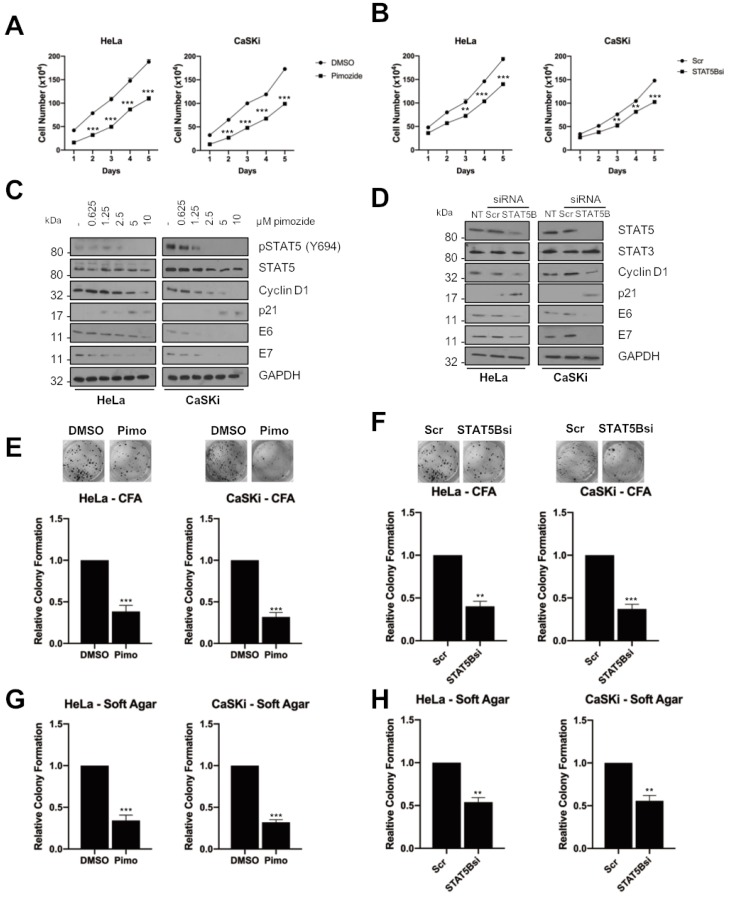
STAT5 is required for proliferation in HPV+ cervical cancer cells. (**A**) Growth curve analysis of HeLa (left) and CaSKi (right) cells after addition of pimozide for 48 h. (**B**) Growth curve analysis of HeLa (left) and CaSKi (right) after transfection of a pool of four specific STAT5B siRNA for 72 h. (**C**) Representative western blot of pimozide dose response in HeLa and CaSKi cells after 48 h. Densitometry analysis is in Supplementary Figure S6A. (**D**) Representative western blot of HeLa and CaSKi cells after transfection of a pool of four specific STAT5B siRNA for 72 h. Densitometry analysis is in Supplementary Figure S6B. (**E**) Colony formation assay (anchorage dependent growth) of HeLa and CaSKi cells after addition of pimozide for 48 h. (**F**) Colony formation assay (anchorage dependent growth) of HeLa and CaSKi cells after transfection of a pool of four specific STAT5B siRNA for 72 h. (**G**) Soft agar assay (anchorage independent growth) of HeLa and CaSKi cells after addition of pimozide for 48 h. (**H**) Soft agar assay (anchorage independent growth) of HeLa and CaSKi cells after transfection of a pool of four specific STAT5B siRNA for 72 h. Error bars represent the mean ± standard deviation of a minimum of three biological repeats. ** *p* < 0.01, *** *p* < 0.001 (Student’s *t*-test).

STAT5 has previously been shown to be an oncogene in both haematological malignancies and in solid cancers, including squamous cell carcinomas [26,27]. To investigate if there is a role for STAT5 in HPV+ cervical cancer, we firstly used the antipsychotic drug pimozide, which has been demonstrated to inhibit STAT5 [34,35]. In both HeLa and CaSKi cells, inhibition of STAT5, as judged by its phosphorylation at Y694 (Figure 5C), resulted in a significant reduction in cell proliferation (Figure 5A; HeLa, *p* = 0.0004 at day 5; CaSKi, *p* = 6.908 × 10^−5^ at day 5). Figure 5C demonstrates that pimozide resulted in a dose-dependent reduction in STAT5 phosphorylation, as well as a reduction in cyclin D1 expression and an increase in p21 expression. As with JAK2 inhibition, pimozide treatment resulted in a significant decrease in anchorage-dependent (Figure 5E; HeLa, *p* = 0.0002; CaSKi, *p* = 0.003) and anchorage independent colony formation (Figure 5G; HeLa, *p* = 0.0002; CaSKi, *p* = 0.002).

To gain further insight into the role of STAT5 in HPV+ cervical cancer, we assessed the relative expression of the two STAT5 isoforms, *STAT5A* and *STAT5B* [36] For this, we performed reverse-transcription quantitative PCR (RT-qPCR) and in all the HPV+ cervical cancer cells, *STAT5B* was expressed at higher levels when compared to HPV- cervical cancer cells (Supplementary Figure S5A). In contrast, the expression of *STAT5A* was reduced around 70% in all HPV+ cervical cancer cells, consistent with previous work [32]. Having identified STAT5B as the major isoform, we depleted it using a pool of specific siRNAs to confirm the effects observed with pimozide. Depletion of STAT5B led to ~70% reduction in *STAT5B* mRNA, without effecting *STAT5A* levels (Supplementary Figure S5B). Consistent with the small molecule inhibitor data, STAT5B depletion also resulted in a significant reduction in cell proliferation (Figure 5B; HeLa, *p* = 0.0002 at day 5; CaSKi, *p* = 0.0002 at day 5), anchorage-dependent (Figure 5F; HeLa, *p* = 0.03 at day 5; CaSKi, *p* = 0.02 at day 5) and anchorage independent colony formation (Figure 5H; HeLa, *p* = 0.01; CaSKi, *p* = 0.003). STAT5B depletion also reduced cyclin D1 expression and increased p21 expression (Figure 5D), confirming a role for STAT5 in HPV+ cervical cancer cell proliferation.

### 2.6. STAT5 Is Required for HPV+ Cervical Cancer Cell Survival

**Figure 6:**
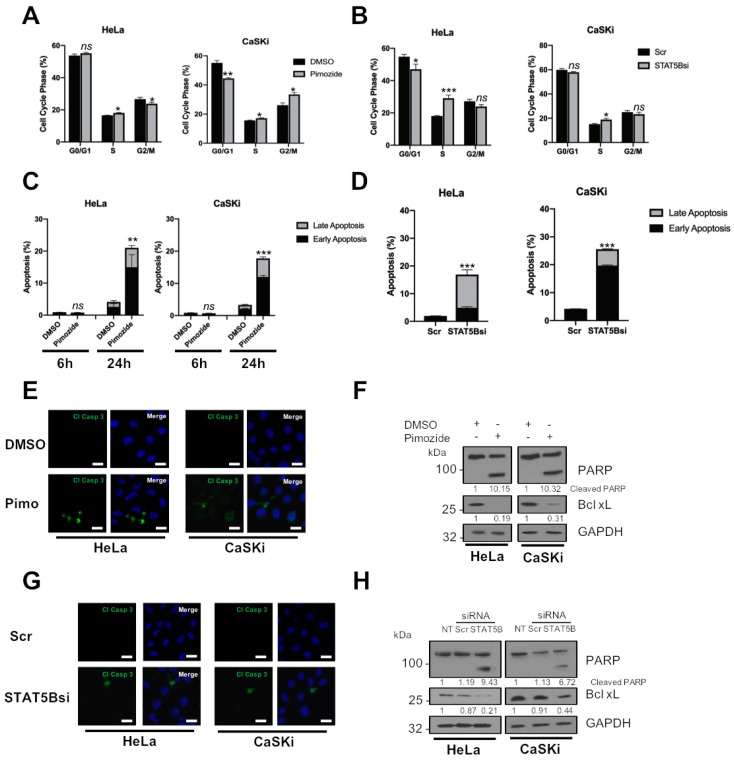
STAT5 is required for HPV+ cervical cancer cell survival. (**A**) Flow cytometric analysis of cell cycle profile of HeLa and CaSKi cells after addition of pimozide for 24 h. (**B**) Flow cytometric analysis of cell cycle profile of HeLa and CaSKi cells after transfection of a pool of four specific STAT5B siRNA for 72 h. (**C**) Flow cytometric analysis of Annexin V assay in HeLa and CaSKi cells after addition of pimozide for 6 and 24 h. (**D**) Flow cytometric analysis of Annexin V assay in HeLa and CaSKi cells after transfection of a pool of four specific STAT5B siRNA for 72 h. (**E**) Immunofluorescence analysis of HeLa and CaSKi cells after addition of pimozide for 6 h. Cover slips were stained for Cleaved caspase 3 (green). Nuclei were visualised using DAPI (blue). Scale bar 20 μM. (**F**) Representative western blot of HeLa and CaSKi cells after addition of pimozide for 24 h and analysed for PARP cleavage and Bcl xL expression. GAPDH served as the loading control. Densitometry analysis is provided below each blot. (**G**) Immunofluorescence analysis of HeLa and CaSKi cells after transfection of a pool of four specific STAT5B siRNA for 72 h. Cover slips were stained for Cleaved caspase 3 (green). Nuclei were visualised using DAPI (blue). Scale bar 20 μM. (**H**) Representative western blot of HeLa and CaSKi cells after transfection of a pool of four specific STAT5B siRNA for 72 h and analysed for PARP cleavage and Bcl xL expression. GAPDH served as the loading control. Densitometry analysis is provided below each blot. Error bars represent the mean ± standard deviation of a minimum of three biological repeats. ns- not significant, * *p* < 0.05, ** *p* < 0.01, *** *p* < 0.001 (Student’s *t*-test).

To explore whether STAT5 is required for cell survival, we analysed the effect of STAT5 inhibition or STAT5B depletion on cell cycle progression and apoptosis. In contrast to JAK2 inhibition, STAT5 inhibition resulted in an increase in cells in the S phase of the cell cycle (Figure 6A; HeLa G0/G1, *p* = 0.017; CaSKi G0/G1, *p* = 0.035), similar to that observed upon STAT3 inhibition. Again, similar results were obtained when STAT5B was depleted using siRNA (Figure 6B; HeLa G0/G1, *p* = 0.0002; CaSKi G0/G1, *p* = 0.05).

We next looked at the effect of STAT5 on cell survival and, although pimozide treatment had little effect on apoptosis after 6 h, by 24 h, there was a significant increase in both early and late apoptosis in both HeLa and CaSKi cells (Figure 6C; total apoptosis at 24 h, HeLa *p* = 0.004; CaSKi *p* = 1.78 × 10^−5^). Similar results were obtained when STAT5B was depleted using STAT5B siRNA (Figure 6D; total apoptosis at 24 h, HeLa, *p* = 0.00059; CaSKi, *p* = 9.9878 × 10^−7^).

As with our JAK2 data, STAT5 inhibition or STAT5B depletion resulted in the activation of caspase 3 activity (Figure 6E,F), PARP cleavage and reduced expression of Bcl-_XL_ (Figure 6G,H), suggesting that STAT5 inhibition or depletion of STAT5B results in the induction of apoptosis.

### 2.7. Phosphorylated JAK2 Positively Correlates with STAT3 and STAT5 Phosphorylation in Cervical Disease and HPV+ Cervical Cancer Cells

Having shown both STAT3 and STAT5 are substrates for JAK2 in HPV+ cervical cancer cells, it was important to understand if the phosphorylation and activation of these proteins correlated during disease progression. Data from Figure 1 and Figure 4 for JAK2 and STAT5, and from our previous published work for STAT3 [21], demonstrate that increases in the phosphorylation of JAK2, STAT3 and STAT5 correlates with cervical disease progression. Furthermore, whereas total JAK2 expression remains unchanged, STAT3 and STAT5 expression also increases upon disease progression.

**Figure 7:**
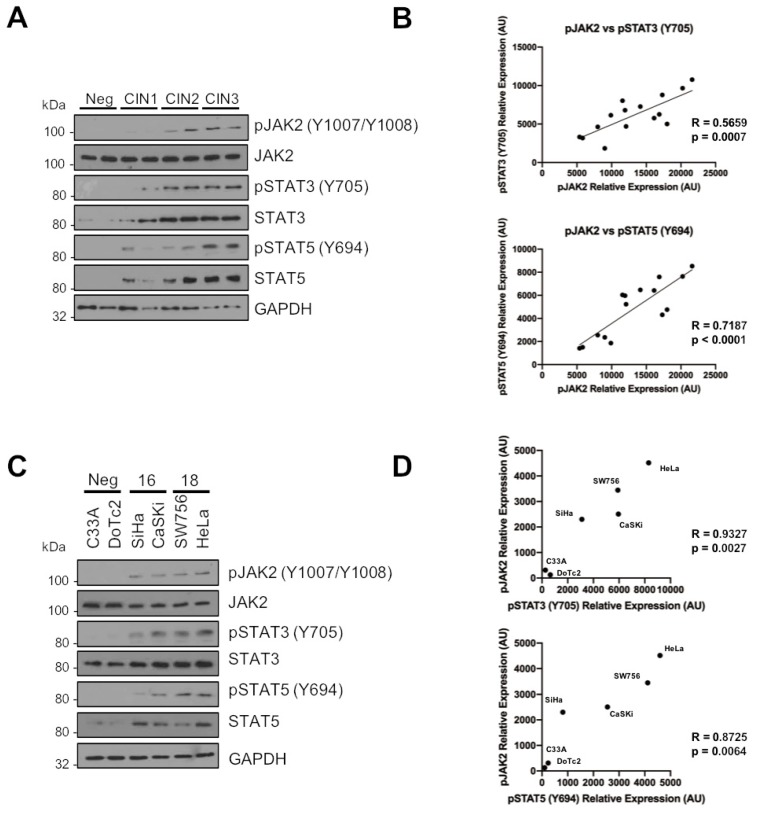
Phosphorylated JAK2 positively correlates with STAT3 and STAT5 phosphorylation in cervical disease and HPV+ cervical cancer cells. (**A**) Representative western blots from cytology samples of CIN lesions of increasing grade analysed for phosphorylated and total JAK2, STAT3 and STAT5 expression. GAPDH served as a loading control. (**B**) Scatter dot plot of densitometry analysis of a panel of cytology samples. Samples from CIN 3 were analysed by western blot and densitometry analysis was performed using ImageJ. Graphs represent the correlation between phosphorylated JAK2 and both phosphorylated STAT3 and STAT5 from matched cytology samples. (**C**) Representative western blot of from six cervical cancer cell lines—two HPV- (C33A and Dotc2 4510), two HPV16+ (SiHa and CaSKi) and HPV18+ (SW756 and HeLa)—for the expression of phosphorylated and total JAK2, STAT3 and STAT5. GAPDH served as a loading control. (**D**) Scatter Dot plot show correlation between phosphorylated JAK2 and both phosphorylated STAT3 and STAT5 in cervical cancer cell lines.

To look at the correlation of JAK2, STAT3 and STAT5 phosphorylation in cervical disease, we plotted the normalized intensities of phosphorylated JAK2 against that of phosphorylated STAT3 and phosphorylated STAT5 from CIN 3, which represents pre-cancerous lesions [37]. The data show that both STAT3 phosphorylation and STAT5 phosphorylation correlate with JAK2 phosphorylation (Figure 7A, quantified for CIN 3 in Figure 7B, pJAK2/pSTAT3 R = 0.5659, *p* = 0.0007; pJAK2/pSTAT5 R = 0.7187, *p* < 0.0001), suggesting a functionally relevant correlation between JAK2 activation and phosphorylated STAT3 and STAT5. Moreover, JAK2 phosphorylation correlated with STAT3/STAT5 phosphorylation in HPV+ cervical cancer cell lines (Figure 7C, pJAK2/pSTAT3 R = 0.9327, *p* = 0.0017; pJAK2/pSTAT5 R = 0.8725, *p* = 0.0064), together suggesting that JAK2 functionally correlates with STAT3/STAT5 phosphorylation in HPV+ cervical cancer.

### 2.8. JAK2 Inhibition Sensitises HPV+ Cervical Cancer Cells to Cisplatin-Induced Apoptosis

**Figure 8:**
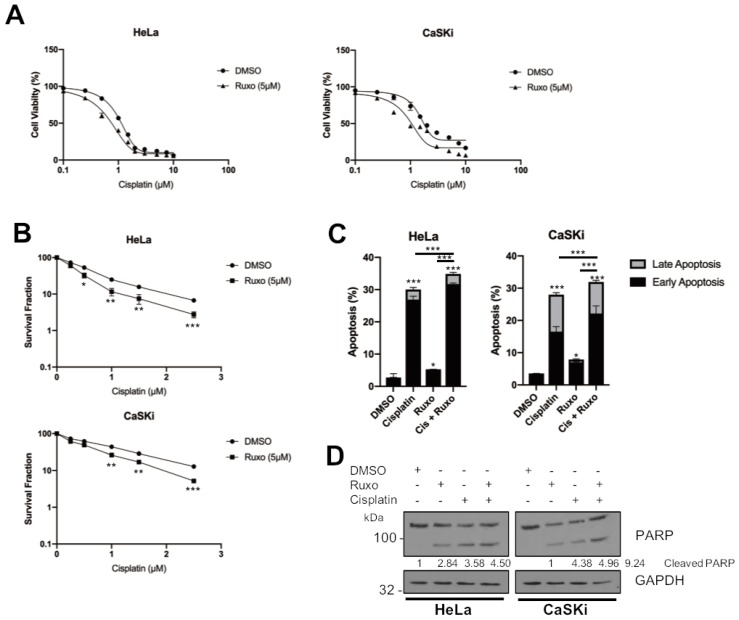
JAK2 inhibition sensitises HPV+ cervical cancer cells to cisplatin-induced apoptosis. (**A**) Cell viability assay (MTT) analysis of HeLa and CaSKi cells treated with increasing doses of cisplatin, with or without ruxolitinib (5 μM), for 24 h. (**B**) Clonogenic survival assay of HeLa and CaSKi cells treated with increasing doses of cisplatin, with or without ruxolitinib (5 μM), for 24 h. (**C**) Flow cytometric analysis of Annexin V assay in HeLa and CaSKi cells after addition of DMSO, ruxolitinib (5 μM), cisplatin (2 μM) or both ruxolitinib and cisplatin for 24 h. (**D**) Representative western blot of HeLa and CaSKi cells after addition of DMSO, ruxolitinib (5 μM), cisplatin (2 μM) or both ruxolitinib and cisplatin for 24 h and analysed for PARP cleavage GAPDH served as the loading control. Densitometry analysis is provided below each blot. Error bars represent the mean ± standard deviation of a minimum of three biological repeats. * *p* < 0.05, ** *p* < 0.01, *** *p* < 0.001 (Student’s *t*-test).

The activation of STAT3 is a common mechanism of resistance to many chemotherapeutics [38]. Previous studies have shown that STAT3 inhibition can sensitize cervical cancer to growth inhibition and apoptosis induced by cisplatin, a common therapeutic used to treat these cancers [39,40] Furthermore, ruxolitinib has also been demonstrated to synergise with cisplatin in HNSCC and non-small cell lung carcinoma (NSCLC) [41,42]. We therefore assessed if the inhibition of JAK2 using ruxolitinib could synergise with cisplatin in HPV+ cervical cancer. Cervical cancer cells were treated with increasing doses of cisplatin with or without ruxolitinib (5 μM) and cell viability was analysed by MTT assay. Alone, cisplatin treatment resulted in a dose-dependent reduction in cell viability (Figure 8A, HeLa, IC_50_ = 0.96 μM; CaSKi, IC_50_ = 1.48 μM) and combination treatment with ruxolitinib enhanced the cisplatin induced cytotoxicity in these cells (Figure 8A, HeLa, IC_50_ = 0.48 μM; CaSKi, IC_50_ = 0.8 μM). Additionally, we performed a clonogenic survival assay, in which cells seeded at a low confluency were treated with increasing doses of cisplatin with or without ruxolitinib (5 μM) and left to grow for 14 days. Cisplatin treatment led to a dose dependent decrease in colony formation (Figure 8B). Furthermore, the addition of ruxolitinib sensitised HPV+ cervical cancers cells to cisplatin, as indicated by a reduction in the survival fraction. These data further demonstrated that ruxolitinib sensitises HPV+ cervical cancer cells to cisplatin induced cytotoxicity and inhibition of cell proliferation (Figure 8B). Finally, the addition of ruxolitinib resulted in an enhancement of apoptosis induced by cisplatin when assessed using the Annexin V assay and PARP cleavage (Figure 8C,D). Together, we demonstrated that ruxolitinib synergises with cisplatin in the induction of apoptosis in HPV+ cervical cancer cells and may be a therapeutic option in these cancers.

## 3. Discussion

The transcription factor STAT3 is activated in ~70% of haematological and solid tumours, including HNSCC, glioblastoma and pancreatic cancer [11,43,44]. STAT3 controls the expression of genes that contribute to the hallmarks of cancer, including proliferation, survival, angiogenesis, immune evasion and inflammation [45]. In cervical cancer, STAT3 has previously been shown to be required for viral oncogene expression and for cell growth [46,47]. Our previous studies demonstrate that STAT3 is also required during the viral lifecycle by regulating viral gene expression and genome amplification [21]. Furthermore, we showed that STAT3 depletion or inhibition resulted in a proliferation defect and the induction of apoptosis in HPV+ cervical cancer cells [20].

Considerable interest exists in the potential therapeutic benefits of targeting STAT3. This is mainly due to the ability to generate potent, specific STAT3 inhibitors and the number of cancers in which aberrant STAT3 signalling is a major driver of malignancy [12,48,49]. However, a major challenge in the development of clinically tractable STAT3 inhibitors has been toxicity observed in early stage clinical trials [19]. Therefore, an alternative approach to impair STAT3 activity may be to target upstream factors in the hope of reducing toxicities associated with STAT3 inhibitors. JAK2 is predominantly responsible for phosphorylating and activating STAT3. Importantly, the levels of JAK2 autophosphorylation and activation are increased in many cancers, particularly those of a haematopoietic origin. Several of these cancers harbour the JAK2 V617F mutant, which drives cell proliferation and can enable immune escape [50,51,52]. Inhibition of JAK2 using the non-selective compound AG490 has demonstrated that JAK2 activity is required for STAT3 phosphorylation and viral oncogene expression in HPV+ cervical cancer cells [47]. However, this inhibitor has been shown to be poorly specific for JAK kinases, with off target effects including the inhibition of EGFR, a known oncogene in cervical cancer [53]. To demonstrate that JAK2 inhibition is detrimental to HPV+ cervical cancer cells, we utilised the potent, well-characterised JAK1/2 inhibitor ruxolitinib, which has been approved for the treatment of Polycythemia Vera (PV) [22], and the JAK2 inhibitor fedratinib, which is currently in Phase III trials for myeloproliferative disorders, were utilised to inhibit JAK2 in HPV+ cervical cancer. In these cells, use of either inhibitor resulted in a significant inhibition in cell proliferation (Figure 2A) and colony formation (Figure 2E,G). JAK2 inhibition also decreased STAT3 tyrosine phosphorylation and reduced the expression of the STAT3 target gene cyclin D1, with a corresponding increase in p21 expression. JAK2 was also required for cell survival, possibly through the reduction of pro-survival proteins such as Bcl xL; therefore, the mechanism of apoptosis induction upon JAK2 inhibition may be due to the reduction in STAT3 dependent Bcl-_XL_ expression. Interestingly, the inhibitors used in this study had slightly different effects depending on the HPV type of the cell lines used. This could potentially mean that there could be different patient responses to any treatments utilizing these inhibitors depending on the HPV type of the tumour; however, more detailed and expansive studies are required to investigate this observation. Together, these data demonstrate the importance of JAK2 mediated signalling in driving the proliferation of HPV+ cervical cancer cells by using highly specific, clinically validated small molecule inhibitors.

JAK2 inhibition also resulted in an arrest of the cell cycle and the induction of apoptosis in HPV+ cervical cancer cells. However, whereas STAT3 inhibition or depletion resulted in an accumulation of cells in the S phase of the cell cycle, JAK2 inhibition resulted in an accumulation of cells in G0/G1 (Figure 3A,B). This is likely due to the fact that JAK2 feeds into other signalling pathways besides STAT3, including STAT5 [54], MAPK [55] and PI3K/AKT [56]. We therefore looked at the effect of JAK2 inhibition or depletion on STAT5 signalling, another STAT family member which has been demonstrated to be activated during the HPV life cycle [30]. Depletion of STAT5 activity had similar effects to JAK2 inhibition; use of the small molecule inhibitor pimozide or depletion of STAT5B using siRNA resulted in a similar proliferation defect and the induction of apoptosis. However, the targeting of STAT5 led to an S-phase block in the cell cycle, similar to the effect of STAT3 inhibition and in contrast to the effects of JAK2 inhibition; these data suggest that other JAK2 substrates may be responsible for the G1-arrest seen in JAK2 inhibited cells. JAK2 has previously been shown to directly phosphorylate the cyclin-dependent kinase inhibitor protein p27^Kip1^, resulting in a G1-arrest [24]. Therefore, further studies are required in order to investigate the potential downstream targets of JAK2 that may be involved in cell cycle progression in cervical cancer cells.

Interestingly, the inhibition or depletion of JAK2 and STAT5B also resulted in a reduction in the expression of the HPV oncoproteins E6 and E7 (Figure 2C). This was similar to our previous study, which demonstrated that inhibition or depletion of STAT3 resulted in a similar reduction of these proteins. These data suggest that the signalling pathways involving JAK2/STAT3 and JAK2/STAT5B are involved in the regulation of viral protein expression. In line with this, a previously study suggested that STAT3 can bind to the viral upstream regulatory region (URR) of HPV16, driving E7 expression [57]. However, our studies in HPV-containing primary keratinocytes did not demonstrate direct binding of STAT3 to the HPV18 URR [21]. Therefore, further studies are required to assess the mechanistic basis of viral protein expression regulated by this family of transcription factors and whether or not JAK2 signalling regulates HPV E6/E7 transcription via other cellular factors.

To gain more information into the correlation of these signalling pathways in cervical disease, we used liquid cytology samples of different grades of cervical disease. The data shows that JAK2, STAT3 and STAT5 phosphorylation correlates with cervical disease progression. Further JAK2 phosphorylation also correlates with both STAT3 and STAT5 phosphorylation, suggesting that the JAK2/STAT3 and JAK2/STAT5 pathways are functional in these cancer cells. This, together with our small molecule inhibitor and siRNA data, demonstrate that targeting these pathways may offer a therapeutic avenue in these cancers. Further studies will be required to assess if the use of clinically available JAK inhibitors such as ruxolitinib offer therapeutic benefit in vivo.

One of the most common treatments for cervical cancer is platinum-based chemotherapeutics, such as cisplatin. One common mechanism of cisplatin resistance in cancer is the activation of STAT3, including in cervical cancer [38]. We therefore rationalised that inhibition of STAT3 using the clinically approved JAK2 inhibitor ruxolitinib may synergise with cisplatin in inducing cell death in HPV+ cervical cancer cells. Our data demonstrate that ruxolitinib and cisplatin alone inhibit proliferation and induce apoptosis; however, in combination, this effect is enhanced, suggesting that these treatments are able to synergise in inducing apoptosis in HPV+ cervical cancer cells.

## 4. Materials and Methods

### 4.1. Cervical Cytology Samples

Cervical cytology samples were obtained from the Scottish HPV Archive (http://www.shine/mvm.ed.ac.uk/archive.shtml), a biobank of over 20,000 samples designed to facilitate HPV associated research. The East of Scotland Research Ethics Service has given generic approval to the Scottish HPV Archive as a Research Tissue Bank (REC Ref 11/AL/0174) for HPV related research on anonymised archive samples. Samples are available for the present project though application to the Archive Steering Committee (HPV Archive Application Ref 0034). RNA and protein were extracted from the samples using Trizol as described by the manufacturer (ThermoFischer Scientific, Waltham, MA, USA) and analysed as described.

### 4.2. Cell Culture

C33A (HPV negative cervical carcinoma), DoTc2 4510 (HPV negative cervical carcinoma), SiHa (HPV16 positive cervical squamous carcinoma), CaSKi (HPV16 positive cervical squamous carcinoma), SW756 (HPV18 positive squamous carcinoma) and HeLa (HPV18 positive cervical epithelial adenocarcinoma) cells were purchased from ATCC and grown in Dulbecco’s modified Eagle’s media (DMEM) supplemented with 10% Foetal Bovine Serum (FBS) (ThermoFischer Scientific, Waltham, MA, USA) and 50 U/mL penicillin/streptomycin (Lonza, Basel, Switzerland).

### 4.3. Small Molecule Inhibitors

The JAK1/2 inhibitor ruxolitinib was purchased from Calbiochem, part of Merckm Darmstadt, Germany) and used at a final concentration of 10 μM unless otherwise stated. The JAK2 inhibitor fedratinib was a kind gift from Dr. Edwin Chen (University of Leeds, Leeds, UK) and used at a final concentration of 10 μM unless otherwise stated. The antipsychotic drug pimozide was purchased from Calbiochem and used at a final concentration of 10 μM unless otherwise stated. Cisplatin was purchased from Cambridge Bioscience and used at a concentration of 2 μM, unless otherwise stated.

### 4.4. siRNA Reagents

For siRNA experiments, four individual siRNA sequences specifically targeting JAK2 or STAT5B were purchased from Qiagen. For each experiment, 50 nM of pooled siRNA was used cells were transfected using lipofectamine 2000 (1:2 ratio) and cell lysates were harvested after 72 h.

### 4.5. Transfections and Mammalian Cell Lysis

Transient transfections were performed with a DNA to Lipofectamine 2000 (ThermoFischer Scientific, Waltham, MA, USA) ratio of 1:2.5. 48 h post transfection, cells were lysed in Leeds lysis buffer for western blot [21].

### 4.6. Western Blotting

Total protein was resolved by SDS Polyacrylamide gel electrophoresis (SDS-PAGE) (10–15% Tris-Glycine), transferred onto Hybond nitrocellulose membrane (Amersham biosciences, Little Chalfont, UK) and probed with antibodies specific for phospho-STAT3 (S727) (ab32143, Abcam, Cambridge, UK), phospho-STAT3 (Y705) (9131, Cell Signalling Technology, Danvers, MA, USA (CST)), STAT3 (124H6:9139, CST), phospho-STAT5 (Y694) (D47E7; 4322, CST), STAT5 (D3N2B; 25656, CST), Phospho-JAK2 (Y1007/1008) (3776, CST), Total JAK2 (3230, CST), HPV18 E6 (G-7, Santa Cruz Biotechnology (SCBT)), HPV18 E7 (8E2, Abcam (ab100953), HPV 16/18 E6 (ab70, abcam), HPV 16 E7 (ED17, SCBT), Cyclin D1 (ab134175, Abcam), p21 (2947, CST), GAPDH (G-9, SCBT), PARP-1 (9542, CST) and Bcl-_xL_ (2764, CST). Western blots were visualized with species-specific HRP conjugated secondary antibodies (Sigma, St. Louis, MO, USA) and ECL (Thermo/Pierce). Densitometry analysis was performed using ImageJ analysis software (NIH, Bethesda, MD, USA) and is provided either in the main figures or as Supplementary Material. Expanded, uncropped western blot panels are also provided as Supplementary Material. Detailed information can be found at Supplementary Figures S7–S9.

### 4.7. Cell Viability Assay

MTT assay to assess cell viability was performed according to the manufacturer’s instructions (Sigma, St. Louis, MO, USA).

### 4.8. Colony Formation Assay

Cells were treated or transfected as required. 48 h post-transfection, cells were trypsinised and reseeded in a six well plate at 500 cells per well and left to incubate for 14–21 days. Colonies were then stained (1% crystal violet, 25% methanol) and colonies were counted manually. Each experiment was repeated a minimum of three times.

### 4.9. Soft Agar Assay

Cells were treated or transfected as required. 60 mm dishes were coated with a layer of 1% agarose (ThermoFischer Scientific, Waltham, MA, USA) in 2× DMEM (ThermoFischer Scientific, Waltham, MA, USA) supplemented with 20% FBS. 48 h post-transfection, cells were trypsinised and added to 0.7% agarose in 2× DMEM (ThermoFischer Scientific, Waltham, MA, USA) supplemented with 20% FBS at 1000 cells/mL. Once set, DMEM supplemented with 10% FBS and 50 U/mL penicillin was added. The plates were then incubated for 14–21 days. Each experiment was repeated at least three times. Visible colonies were counted manually.

### 4.10. Clonogenic Survival Assay

Cells were treated with increasing doses of cisplatin in the presence of DMSO or 5 μM ruxolitinib. After 24 h, cells were trypsinised and reseeded in a six well plate at different densities depending on the cisplatin dose used. Cells were left to incubate for 14 days. Colonies were then stained (1% crystal violet, 25% methanol) and colonies were counted manually. The plating efficiency and survival fraction was calculated according to [58]. Each experiment was repeated a minimum of three times.

### 4.11. Annexin V Assay

Annexin V apoptosis assay (TACS Annexin V kit; 4830-250-K) was performed as indicated on the product datasheet. Briefly, cells were seeded in 6-well plates at a density of 1 × 10^6^ cells/mL and were treated as required per experiment. Cells were then trypsinised and collected by centrifugation at 700× *g* for 5 min. Cells were then washed in cold PBS and re-centrifuged. 1 × 10^6^ cells were then incubated in 100 μL Annexin V reagent (10 μL 10× binding buffer, 10 μL propidium iodide, 10 μL Annexin V-FITC (diluted 1 in 250) and 880 μL ddH_2_O) for 15 min at room temperature in the dark. 400 μL of 1× binding buffer was then added before analysis by flow cytometry. Samples were processed on an LSRFortessa^TM^ cell analyzer (BD) and the PI histograms analysed on modfit software.

### 4.12. Quantitative Real-Time PCR

Total RNA was extracted using the E.Z.N.A. Total RNA Kit I (Omega Bio-Tek) according to the manufacture’s protocol. 1 μg of total RNA was DNase treated following the RQ1 RNase-Free DNase protocol (Promega) and then reverse transcribed with a mixture of random primers and oligo(dT) primers using the qScriptTM cDNA SuperMix (Quanta Biosciences, Beverly, MA, USA) according to instructions. RT-qPCR was performed using the QuantiFast SYBR Green PCR kit (Qiagen, Toronto, ON, Canada). The PCR reaction was conducted on a Corbett Rotor-Gene 6000 (Qiagen) as follows: initial activation step for 10 min at 95 °C and a three-step cycle of denaturation (10 s at 95 °C), annealing (15 s at 60 °C) and extension (20 s at 72 °C) which was repeated 40 times and concluded by melting curve analysis. The data obtained was analysed according to the ΔΔCt method using the Rotor-Gene 6000 software [59]. Specific primers were used for each gene analysed. U6 served as normaliser gene.

### 4.13. Immunofluorescent Staining

Cells were seeded onto coverslips and, 24 h later, were transfected as required. 24 h after transfection, cells were fixed with 4% paraformaldehyde for 10 min and then permeabilised with 0.1% (*v/v*) Triton for 15 min. Cells were then incubated in cleaved caspase 3 (Asp175; 9661, CST) primary antibody in PBS with 4% BSA overnight at 4 °C. Primary antibodies were used at a concentration of 1:400. Cells were washed thoroughly in PBS and then incubated with Alex-fluor conjugated secondary antibodies 594 and Alexa 488 (1:1000) (Invitrogen, part of ThermoFischer Scientific, Waltham, MA, USA) in PBS with 4% BSA for 2 h. DAPI was used to visualise nuclei. Coverslips were mounted onto slides with Prolong Gold (Invitrogen, part of ThermoFischer Scientific, Waltham, MA, USA).

### 4.14. Statistical Analysis

Where indicated, data was analysed using a two-tailed, unpaired Student’s *t*-test.

## 5. Conclusions

In conclusion, we have demonstrated for the first time that the targeting of JAK2 using the clinically available inhibitior ruxolitinib inhibits proliferation in HPV+ cervical cancers cells and induces apoptosis. Furthermore, we show that ruxolitinib may synergise with cisplatin in the induction of cell death in HPV+ cervical cancer cells. The data in this study, and our previous work, demonstrate that JAK2 can activate both STAT3 and STAT5 signalling in HPV+ cervical cancer cells, suggesting that both pathways contribute to the tumourigenesis of these cancers. Together, these results suggest that JAK2 inhibitors may potentially be utilised as combination therapies with currently approved cervical cancer chemotherapeutic, such as cisplatin.

## Supplementary Materials

The following are available online at https://www.mdpi.com/2072-6694/11/12/1934/s1, Figure S1: Effect of JAK2 inhibitors on JAK2 and STAT3 phosphorylation, Figure S2: Inhibition of JAK2 has minimal impact on proliferation in HPV- cervical cancer cells, Figure S3: Densitometry analysis of western blots in Figure 2, Figure S4: Densitometry analysis of western blots in Figure 4, Figure S5: STAT5 isoform expression in cervical cancer cells, Figure S6: Densitometry analysis of western blots in Figure 5, Figure S7: Expanded, uncropped western blot panels from Figure 1, Figure 2, Figure 3 and Figure 4, Figure S8: Expanded, uncropped western blot panels from Figure 5, Figure 6, Figure 7 and Figure 8, Figure S9: Expanded, uncropped western blot panels from Supplementary Figure S1–S3.
