# Supplementary material for: JAK2 Inhibition Impairs Proliferation and Sensitises Cervical Cancer Cells to Cisplatin-Induced Cell Death
Source: Cancers (Basel). 2019 Dec 4;11(12):1934. doi: 10.3390/cancers11121934 (PMC6966458; doi:10.3390/cancers11121934)
Supplement: Supplementary file 1 [file cancers-11-01934-s001.pdf]

Supplementary Materials

JAK2 Inhibition Impairs Proliferation and Sensitises Cervical Cancer Cells to Cisplatin-Induced Cell Death

Ethan L. Morgan and Andrew Macdonald

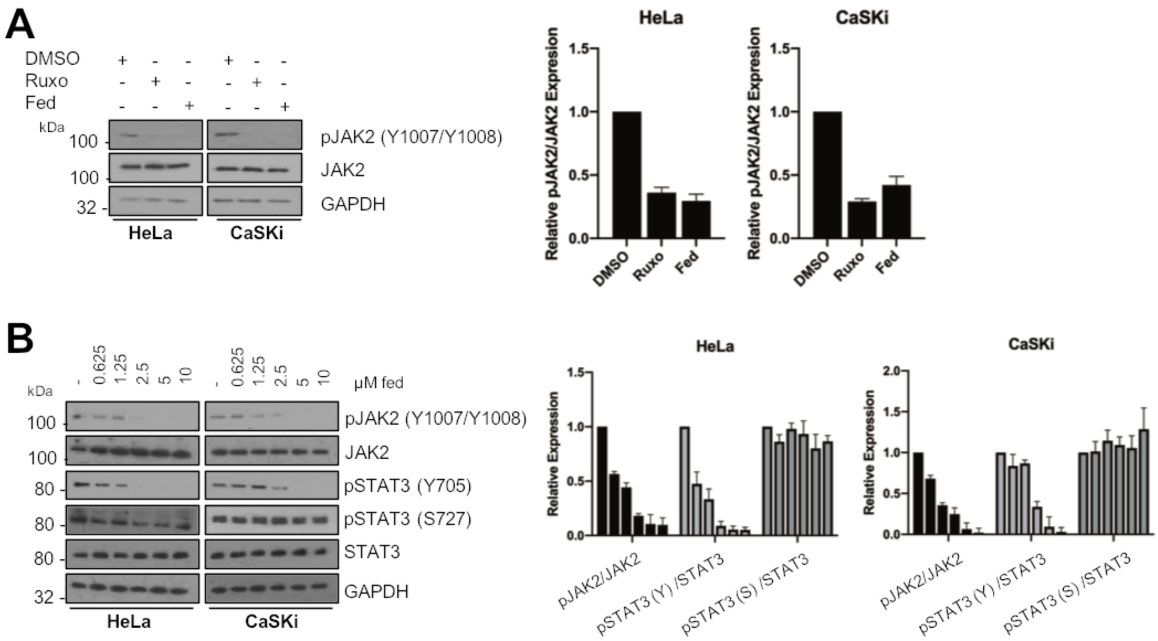

Figure S1. Effect of JAK2 inhibitors on JAK2 and STAT3 phosphorylation. (**A**) (left) Representative western blot of ruxolitinib and fedratinib treatment (10 µM) of HeLa and CaSKi cells after 48 hours. (right) Densitometry analysis of the western blot in A). (**B**) (left) Representative western blot of fedratinib dose response in HeLa and CaSKi cells after 48 hours. (right) Densitometry analysis of the western blot in (**B**).

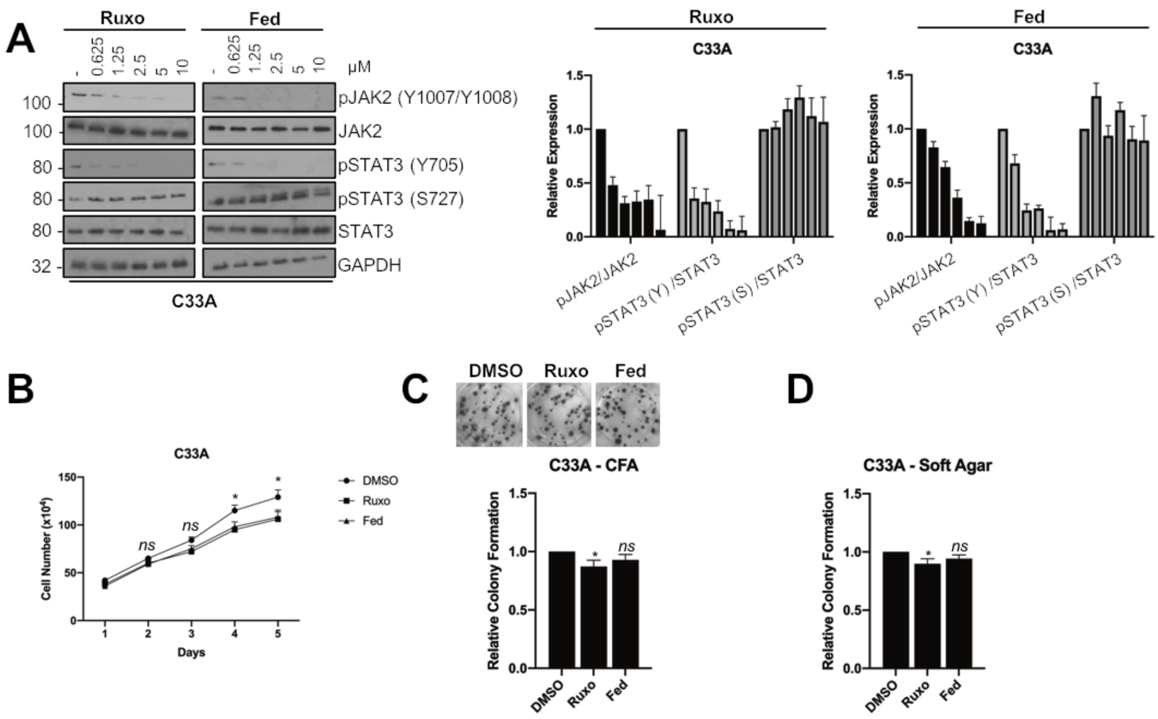

Figure S2. Inhibition of JAK2 has minimal impact on proliferation in HPV- cervical cancer cells. (**A**) (left) Representative western blot of ruxolitinib and fedratinib dose response in HPV- C33A cells. (right) Densitometry analysis of the western blot in A). (**B**) (left) Representative western blot of fedratinib dose response in HeLa and CaSKi cells after 48 hours. (right) Densitometry analysis of the western blot in (**B**). (**B**) Growth curve analysis of HeLa (left) and CaSKi (right) cells after addition of pimozide for 48 hours. (**B**) Growth curve analysis of C33A cells after treatment with ruxolitinib and fedratinib (10 µM) for 48 hours. **C**) Colony formation assay (anchorage dependent growth) of C33A cells after addition of ruxolitinib and fedratinib (10 µM) for 48 hours. **D**) Soft agar assay (anchorage independent growth) of C33A cells after addition of ruxolitinib and fedratinib (10 µM) for 48 hours.Error bars represent the mean Error bars represent the mean +/− standard deviation of a minimum of three biological repeats. ns– not significant, * $p < 0.05$ (Student's *t*-test).

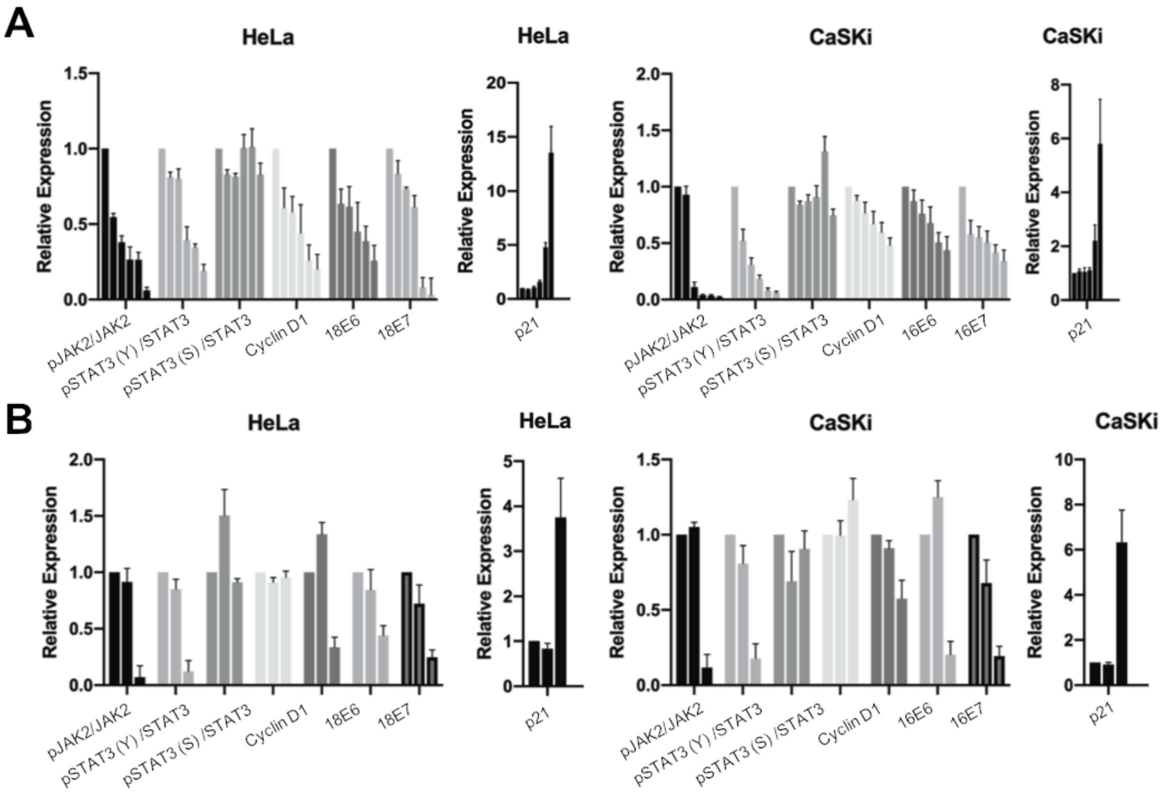

Figure S3. Densitometry analysis of western blots in Figure 2. (**A**) Densitometry analysis of western blots in Figure 2C. (**B**) Densitometry analysis of western blots in Figure 2D. Error bars represent the mean +/− standard deviation of a minimum of three biological repeats.

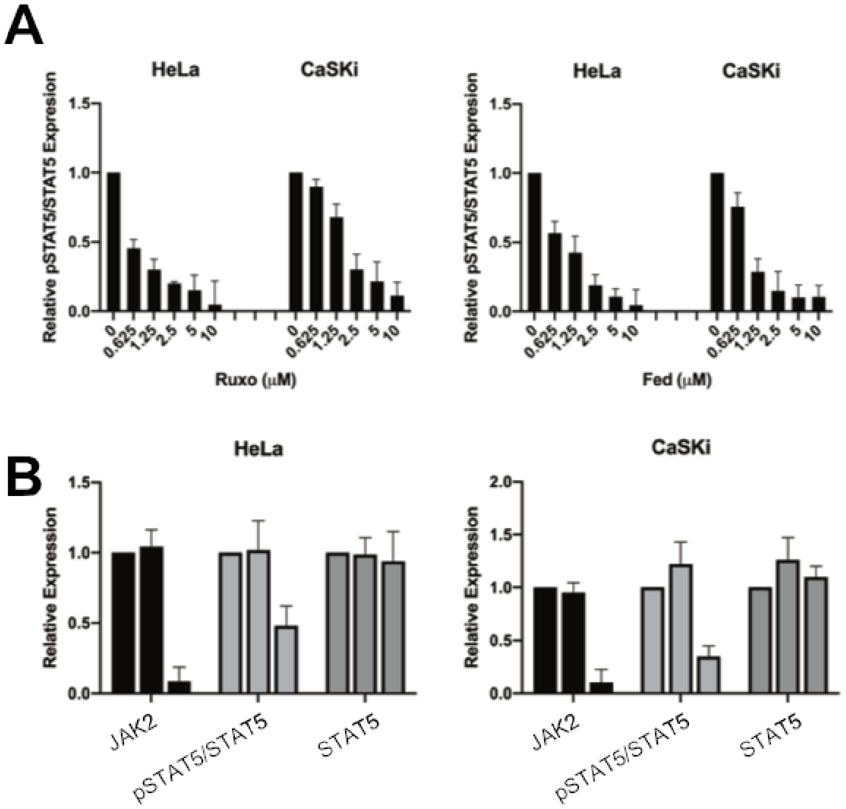

Figure S4. Densitometry analysis of western blots in Figure 4. (**A**) Densitometry analysis of western blots in Figure 4E. (**B**) Densitometry analysis of western blots in Figure 4F. Error bars represent the mean +/− standard deviation of a minimum of three biological repeats.

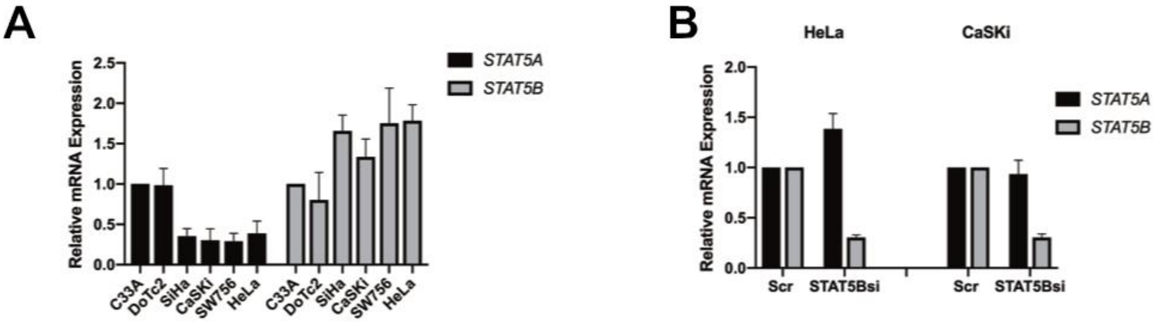

Figure S5. STAT5 isoform expression in cervical cancer cells. (**A**) qPCR analysis of *STAT5A* and *STAT5B* expression in six cervical cancer cell lines—two HPV- (C33A and Dotc2 4510), two HPV16+ (SiHa and CaSKi) and HPV18+ (SW756 and HeLa). (**B**) qPCR analysis of *STAT5A* and *STAT5B* expression in HeLa and CaSKi cells after transfection with a pool of STAT5B specific siRNA. U6 was used as a control. Error bars represent the mean +/− standard deviation of a minimum of three biological repeats.

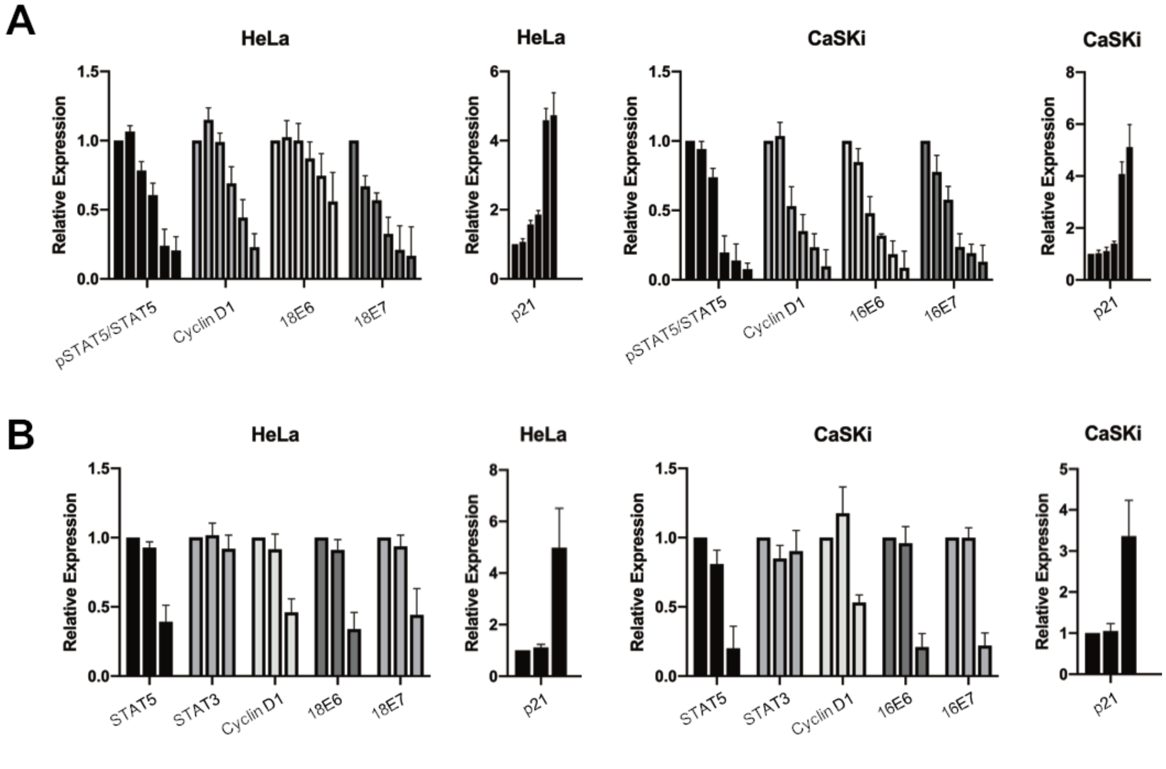

Figure S6. Densitometry analysis of western blots in Figure 5. (**A**) Densitometry analysis of western blots in Figure 5C. (**B**) Densitometry analysis of western blots in Figure 5D. Error bars represent the mean +/− standard deviation of a minimum of three biological repeats.

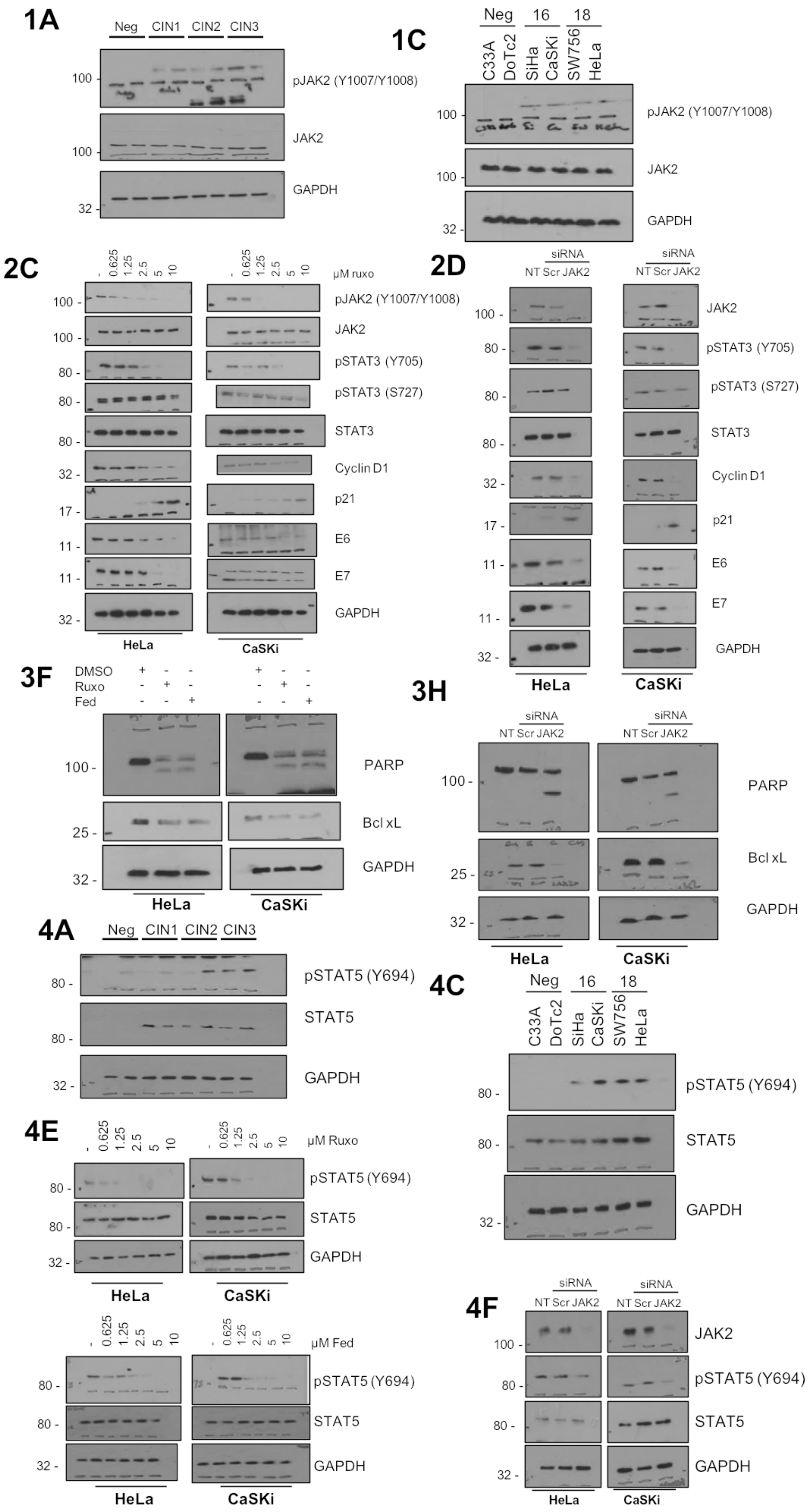

Figure S7. Expanded, uncropped western blot panels from Figures 1–4.

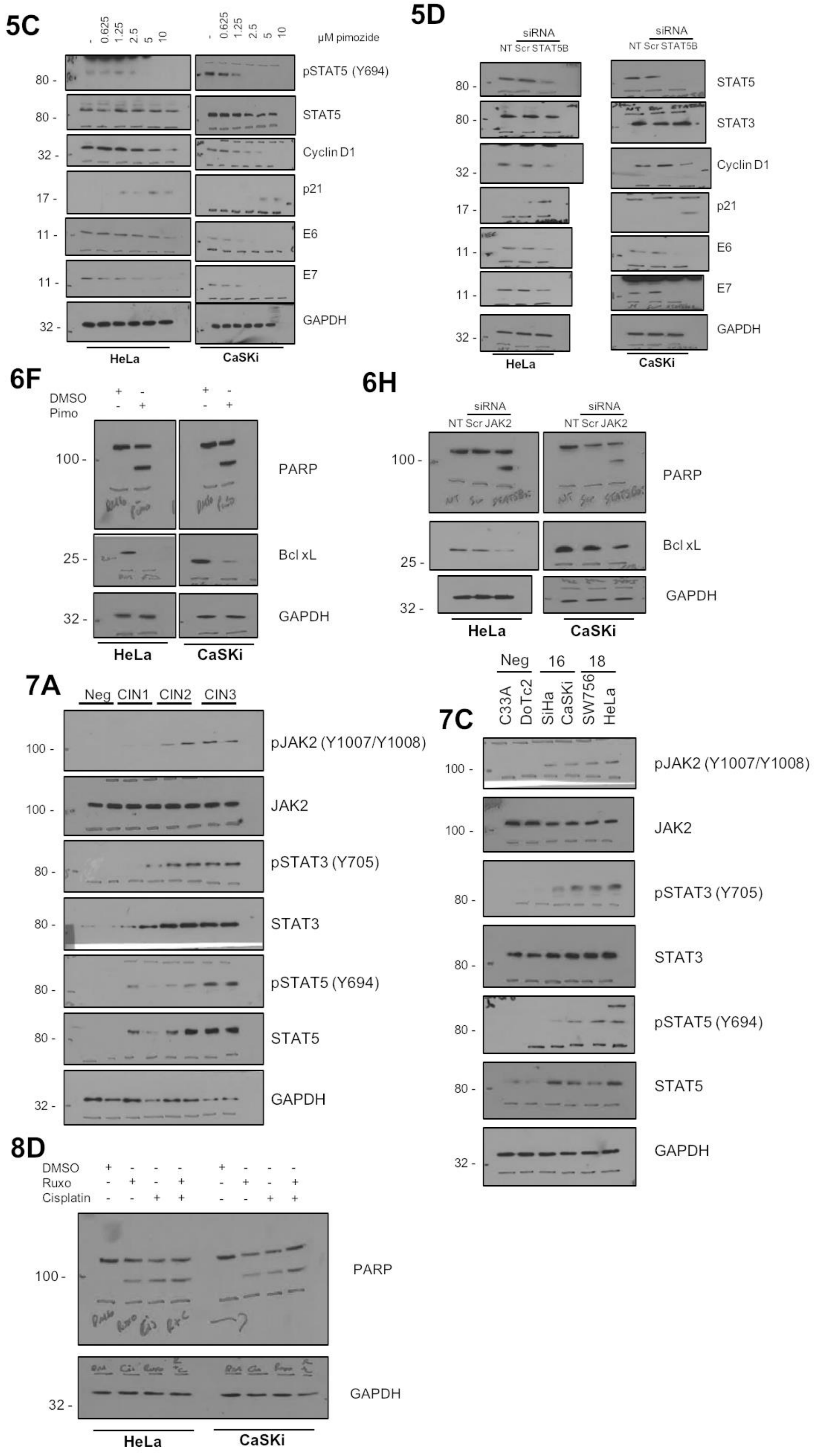

Figure S8. Expanded, uncropped western blot panels from Figures 5–8.

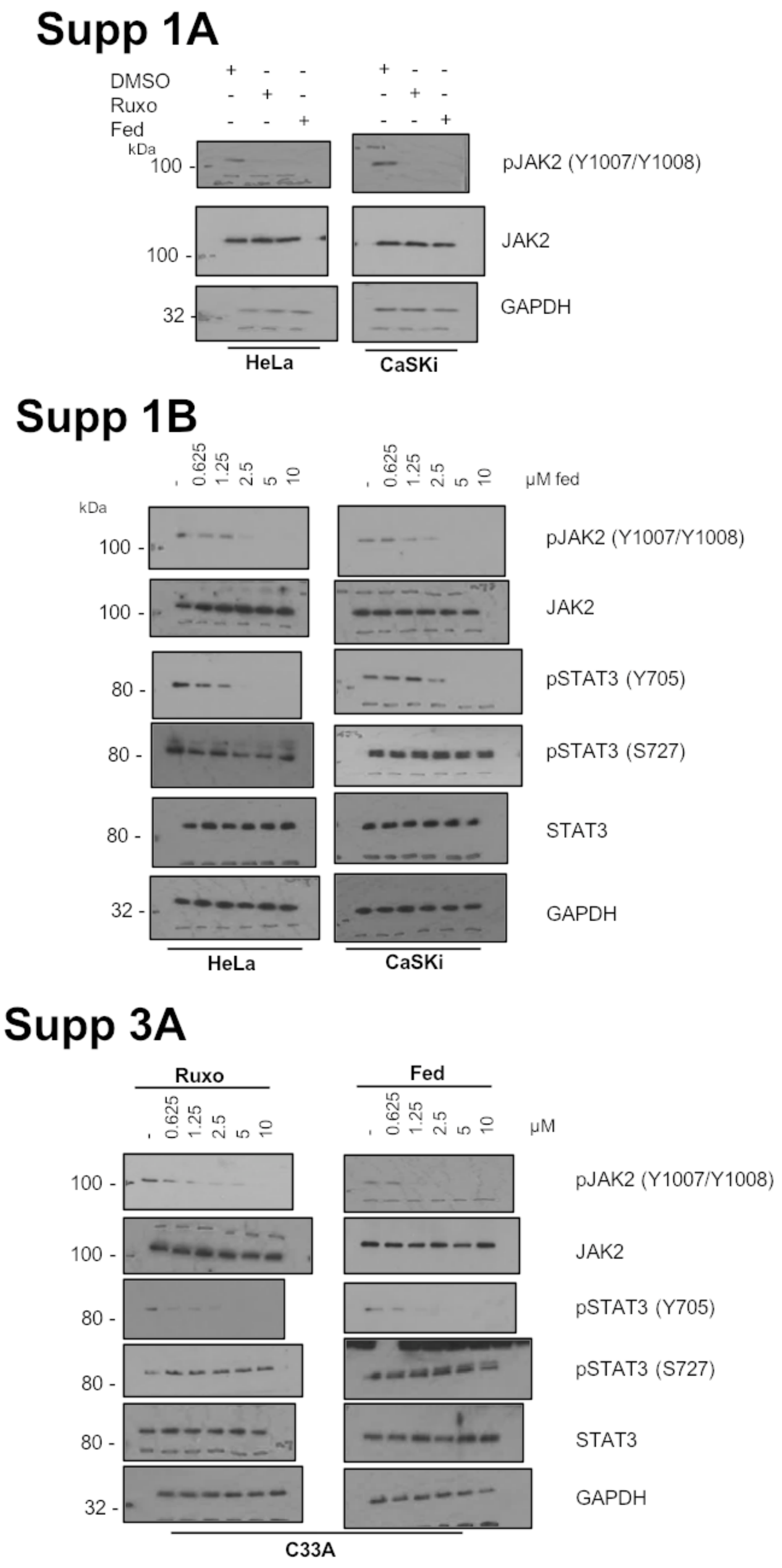

Figure S9. Expanded, uncropped western blot panels from Figures S1–S3.

© 2019 by the authors. Licensee MDPI, Basel, Switzerland. This article is an open access article distributed under the terms and conditions of the Creative Commons Attribution (CC BY) license (http://creativecommons.org/licenses/by/4.0/).
